# Phenotypic diversity of human adipose tissue-resident NK cells in obesity

**DOI:** 10.3389/fimmu.2023.1130370

**Published:** 2023-02-22

**Authors:** Martha E. Haugstøyl, Martin Cornillet, Kristina Strand, Natalie Stiglund, Dan Sun, Laurence Lawrence-Archer, Iren D. Hjellestad, Christian Busch, Gunnar Mellgren, Niklas K. Björkström, Johan Fernø

**Affiliations:** ^1^ Hormone Laboratory, Department of Medical Biochemistry and Pharmacology, Haukeland University Hospital, Bergen, Norway; ^2^ Mohn Nutrition Research Laboratory, Department of Clinical Science, University of Bergen, Bergen, Norway; ^3^ Center for Infectious Medicine, Department of Medicine Huddinge, Karolinska Institutet, Karolinska University Hospital, Stockholm, Sweden; ^4^ Department of Medicine, Haukeland University Hospital, Bergen, Norway; ^5^ Plastikkirurg1, Bergen, Norway

**Keywords:** obesity, adipose tissue inflammation, Natural killer (NK) cell, multicolor flow cytometry, tissue residency, insulin resistance

## Abstract

Natural killer (NK) cells have emerged as key mediators of obesity-related adipose tissue inflammation. However, the phenotype of NK cell subsets residing in human adipose tissue are poorly defined, preventing a detailed understanding of their role in metabolic disorders. In this study, we applied multicolor flow cytometry to characterize CD56^bright^ and CD56^dim^ NK cells in blood and adipose tissue depots in individuals with obesity and identified surface proteins enriched on adipose tissue-resident CD56^bright^ NK cells. Particularly, we found that adipose tissue harbored clusters of tissue-resident CD56^bright^ NK cells signatured by the expression of CD26, CCR5 and CD63, possibly reflecting an adaptation to the microenvironment. Together, our findings provide broad insights into the identity of NK cells in blood and adipose tissue in relation to obesity.

## Introduction

The expansion of adipose tissue in obesity is accompanied by accumulation of pro-inflammatory immune cells and cytokines, causing a state of chronic, low-grade inflammation that may disrupt insulin signaling both in the adipose tissue and systemically (1). Natural killer (NK) cells have been proposed as important mediators of adipose tissue inflammation. NK cells are innate lymphoid cells that can sense different types of stress through the expression of activating and inhibitory receptors, including killer-cell immunoglobulin-like receptors (KIRs), NKG2A, NKG2D and NKp46 (2). Upon activation, NK cells secrete cytotoxic perforin and granzyme molecules to facilitate killing of tumor cells and virus-infected cells. In addition, they secrete cytokines that may influence other immune cells (3). Studies from mice have demonstrated a homeostatic role of NK cells in lean adipose tissue, with increased accumulation and altered phenotype and functionality in obesity (4, 5). Obesity-induced NK cell dysregulations are characterized by increased secretion of interferon (IFN)-γ that can mediate polarization of macrophages towards a pro-inflammatory state. This, combined with reduced NK cell cytotoxicity that hampers their ability to kill specific macrophage populations (6), has been shown to lead to systemic insulin resistance.

In humans, NK cells are broadly divided into two subsets, cytotoxic CD56^dim^CD16^+^ (hereby CD56^dim^) NK cells and cytokine-secreting CD56^bright^CD16^-^ (hereby CD56^bright^) NK cells. In blood, CD56^dim^ NK cells represent around 90% of the total NK cell population, whereas in tissues CD56^bright^ NK cells dominates, where they also express the canonical residency marker CD69. Importantly, the local microenvironment in each tissue contributes to shape unique phenotypic and functional features of these cells, with diverse subset distributions of tissue-resident NK cells present in various tissues with important local immunological functions (7, 8).

Adipose tissue is divided into subcutaneous adipose tissue (SAT) and visceral adipose tissue (VAT). VAT is generally found to harbor more pro-inflammatory immune cells and presents increased risk of developing metabolic disease. In line with these observations, data from murine studies demonstrate higher accumulation of NK cells in VAT compared with SAT in obesity, largely caused by proliferation of tissue-resident NK cells (5, 6, 9). In humans less is known about the distribution and protein signatures of CD56^bright^ and CD56^dim^ NK cells between different adipose tissue depots, and how these features are affected by obesity. Some studies also have investigated NK cells in human adipose tissue (10–13), but the tissue-resident NK cell population is poorly defined, and it is uncertain to what extent these cells display a CD56^bright^ phenotype and whether they can be identified by markers additional to CD69. The lack of information regarding phenotypic markers defining adipose tissue CD56^bright^ and CD56^dim^ NK cells and how they may differ between SAT and VAT, has so far represented a limitation for a detailed understanding of how adipose tissue NK cells may contribute to metabolic disturbances in obesity. Thus, a more comprehensive characterization of these cells is warranted.

In this study, we performed a surface proteome screening to identify novel proteins expressed by NK cells in human adipose tissue. Next, we applied multicolor flow cytometry to evaluate the expression of the novel proteins in combination with established NK cell markers on CD56^bright^ and CD56^dim^ NK cells in blood, SAT, and VAT of a sizeable cohort of individuals with obesity. The findings reveal marked NK cell diversity in adipose tissue and identify multiple clusters that may potentially represent NK cell subsets involved in adipose tissue inflammation.

## Results

### Surface proteome screening reveals distinct proteins expressed by adipose tissue NK cells

**Figure 1:**
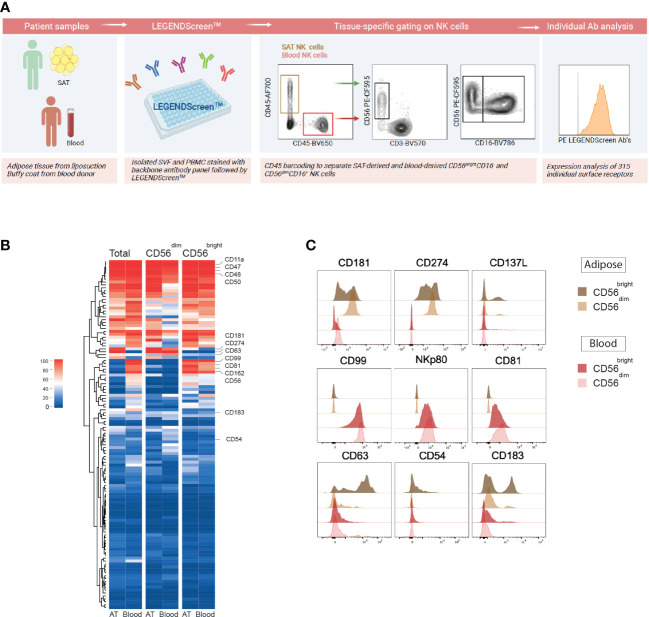
Surface proteome screening of human subcutaneous adipose tissue reveals distinct proteins expressed by adipose tissue NK cells. **(A)** Experimental overview of LEGENDScreen™ surface proteome screening on NK cells in subcutaneous adipose tissue (SAT) and peripheral blood (PBMC). Created with BioRender.com **(B)** Heatmap depicting the percentage of total, CD56^dim^ and CD56^bright^ NK cells expressing 315 individual surface proteins in SAT and PBMC. **(C)** Flow histograms showing a selection of proteins enriched on SAT NK cells (top), blood NK cells (middle) and SAT CD56^bright^ NK cells (bottom).

As a first approach to phenotype NK cells in adipose tissue, we performed LEGENDScreen™ surface proteomic screening to evaluate 315 surface receptors and compare their expression on NK cells in blood (peripheral blood mononuclear cells; PBMCs) and SAT from donors (**Figure 1A**). Substantial overlap in protein expression was observed between total NK cells in blood and adipose tissue, displaying high expression levels of CD11a, CD47, CD48, and CD50 (**Figure 1B**). Several proteins were identified as either blood- or adipose tissue-enriched, such as higher expression of CD181, CD274, and CD137L on SAT NK cells and higher CD99, NKp80, CD81, and GPR56 on blood NK cells (**Figure 1C**). The CD56^bright^ NK cells are normally considered the tissue-resident subset, and to identify potential adipose tissue-resident NK cell markers, we investigated proteins that were more highly expressed by these cells relative to the CD56^dim^ NK cells. Interestingly, levels of several proteins, such as CD63, CD54, and CD183, were expressed by higher proportions of the CD56^bright^ cells in SAT (**Figure 1C**). Taken together, we identified surface proteins enriched on NK cells in blood and adipose tissue, respectively. Moreover, we defined surface proteins characteristic of CD56^bright^ NK cells in adipose tissue that may potentially define adipose tissue-resident NK cell subsets. Of note, the tissue residency marker, CD69, did not emerge as tissue-specific from the proteomic analysis (data not shown), emphasizing certain limitations with this approach and the need to validate our findings.

### Deep-phenotyping of CD56^bright^ and CD56^dim^ NK cells in adipose tissue depots and blood

**Figure 2:**
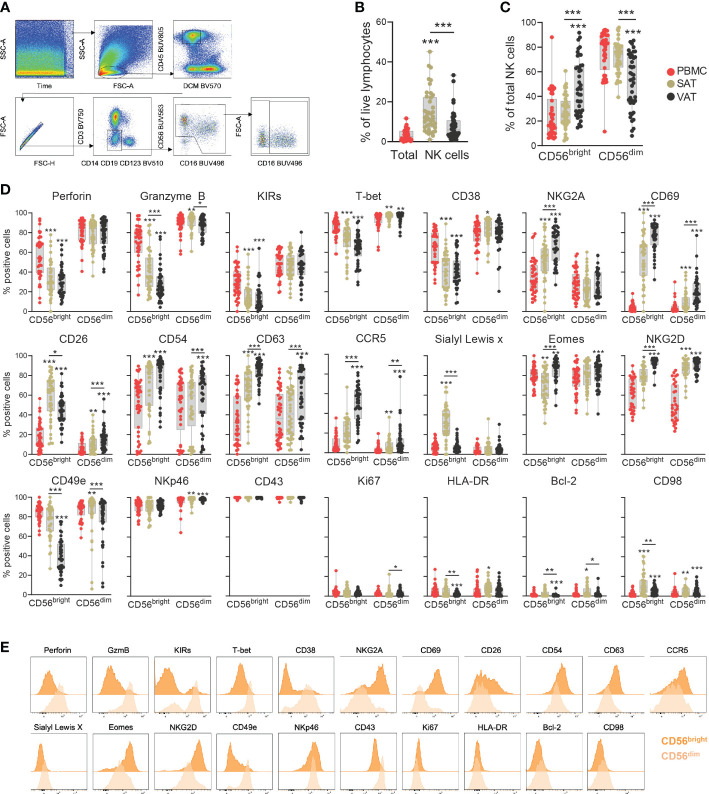
Deep-phenotyping of CD56^bright^ and CD56^dim^ NK cells in adipose tissue depots and blood. **(A)** Gating strategy to identify CD56^bright^ and CD56^dim^ NK cells. Representative VAT sample from one patient. **(B)** Frequency of total NK cells out of viable CD45^+^ lymphocytes and **(C)** Frequencies of CD56^bright^ and CD56^dim^ NK cells out of total NK cells in matched peripheral blood (PBMC), subcutaneous (SAT) and visceral (VAT) adipose tissue of bariatric surgery patients (n=43). **(D)** Box-and-whisker plots depicting the percentage of positive CD56^bright^ and CD56^dim^ NK cells for the individual proteins in PBMC, SAT and VAT. Line represents median, and the whiskers represent the minimum and maximum value. For normally distributed data (CD26, CD38 and NKG2A), one-way ANOVA with Holm-Šídák’s multiple comparisons test was used. When data was not normally distributed, Friedman test with Dunn’s multiple comparisons test was used. *p<0.05, **p<0.01, ***p<0.001. **(E)** Representative staining (expression intensity) of 21 proteins on the NK cell subsets. The plots represent VAT sample from one patient.

To validate the LEGENDScreen™ findings and to further characterize the CD56^bright^ and CD56^dim^ NK cells in blood and adipose tissue, we generated a 27-parameters flow cytometry panel that combined established NK cell-associated markers (e.g., granzyme B, perforin, T-bet, Eomes, KIRs, NKp46, NKG2A, and NKG2D) with the adipose tissue-enriched CD56^bright^ NK cell proteins identified from the screening (CD26, CD54, CD63, CCR5, and Sialyl Lewis^x^). CD69 was also included in the panel as an indicator of tissue residency. The protein expression was measured on CD56^bright^ and CD56^dim^ NK cells from matched blood, SAT, and VAT samples of 43 individuals with obesity, undergoing bariatric surgery (**Table 1**) and the gating strategy is presented in **Figure 2A**.

**Table 1:** Clinical and biochemical parameters.

	Patients (n=43)
Sex (M/F)	13/30
Age (years)	40 (19-65)
T2D	9/43
BMI (kg/m^2^)	38.8 (33.1-51.8)
Fasting glucose (mmol/L)	5.6 (4.4-11.4)
HbA1c (mmol/mol)	36 (29-95)
CRP (mg/L)	5 (1-16)
Insulin (mIU/L)	18.3 (5.2-63.5)
HOMA-IR	4.7 (1.1-18.3)
C-peptide (mmol/L)	1.1 (0.3-2.9)
Total cholesterol (mmol/L)	4.6 (2.5-8.3)
LDL (mmol/L)	3.1 (1.1-6.1)
HDL (mmol/L)	1.1 (0.6-2.0)
TAG (mmol/L)	1.6 (0.5-6.9)

Data are given as median (range). All circulating parameters are measured from fasting blood samples. M, males; F, females; T2D, type 2 diabetes; BMI, body mass index; CRP, C-reactive protein; HOMA-IR, homeostatic model assessment of insulin resistance index; LDL, low-density lipoprotein cholesterol; HDL, high-density lipoprotein cholesterol; TAG; triglycerides.

The proportion of total NK cells was significantly higher in adipose tissue relative to blood and also higher in SAT than in VAT (**Figure 2B**). Similarly, depot-specific differences were found for the relative abundance of CD56^bright^ and CD56^dim^ subsets (**Figure 2C**). As expected, the CD56^bright^ NK cells constituted on average around 10% of the NK cell population in blood, a distribution pattern that was also seen in SAT. In VAT, however, the CD56^bright^ NK cells made up on average almost 50% of the NK cell compartment.

The expression levels of several proteins differed significantly between the CD56^bright^ and CD56^dim^ subsets (**Figures 2D, E**, **Supplementary Figures 1, 2**). As expected, the CD56^dim^ NK cells displayed a cytotoxic- and maturation profile, indicated by consistently higher levels of perforin, granzyme B, T-bet, and KIRs. Interestingly, a proportion of the CD56^bright^ cells was also positive for these markers, suggesting some level of cytotoxic potential for this subset. Additionally, CD56^bright^ cells in the adipose tissue expressed higher levels of NKG2A and CD69, which supports the assumption that CD56^bright^ NK cells represent the major tissue-resident population in adipose tissue. Interestingly, not all CD56^bright^ NK cells were positive for CD69, indicating that there are subsets that do not display classical tissue residency phenotypes (**Figure 2D**). The proteins identified from LEGENDScreen™ also displayed differential expression between the two subtypes, with significantly higher levels of CD26, CD54, CD63, CCR5, and Sialyl Lewis^x^ detected on the CD56^bright^ NK cells in adipose tissue compared with CD56^dim^ NK cells (**Figure 2D**).

Substantial variations in NK cell protein expression were observed between the various tissue compartments (**Figure 2D**). This was particularly evident for the CD56^bright^ subset, of which CD54, CD63, CD69, CCR5, Eomes, and NKG2D, were not only enriched in adipose tissue compared with blood but also significantly higher in VAT than in SAT. Conversely, CD26 and Sialyl Lewis^x^ emerged as SAT-enriched proteins, although CD26 was also expressed by nearly 50% of the CD56^bright^ NK cells in VAT. Perforin, granzyme B, KIRs, T-bet, and CD49e displayed the opposite tissue expression pattern on the CD56^bright^ NK cells, with significantly reduced levels in adipose tissue compared to blood, and lower in VAT than in SAT. The CD56^dim^ NK cells also showed tissue-specific phenotypes, although less striking. Similar to the CD56^bright^ subset, NKG2D was increased on the CD56^dim^ NK cells in adipose tissue compared with blood, also displaying a higher VAT/SAT ratio (**Figure 2D**). Furthermore, a small fraction of the CD56^dim^ NK cells in adipose tissue, particularly in VAT, were positive for CD69, indicating that subsets of CD56^dim^ NK cells also exhibit signs of tissue residency. In summary, our data suggests the existence of tissue-resident CD56^bright^ NK cells enriched in VAT, characterized by the expression of additional surface markers that may potentially reflect adaptations to the microenvironment in this tissue depot.

### NK cell proteins in adipose tissue associate weakly with systemic insulin resistance

**Figure 3:**
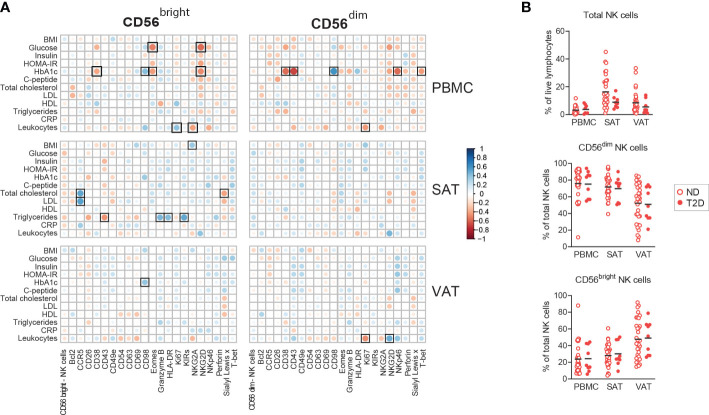
Associations between NK cell proteins and metabolic dysfunction. **(A)** Pearson correlations between NK cell proteins and circulating clinical parameters. Black squares indicate significant at adjusted FDR <0.1. **(B)** Box-and-whisker plots depicting the percentages of total NK cells out of viable CD45^+^ lymphocytes and CD56^bright^ and CD56^dim^ NK cells out of total NK cells from peripheral blood (PBMC), subcutaneous (SAT) and visceral (VAT) adipose tissue from individuals with obesity with type 2 diabetes (T2D, n=9) and without type 2 diabetes (n=34).

Since adipose tissue NK cells are suggested contributors for the development of insulin resistance and metabolic disease, we performed a correlation analysis between the individual proteins expressed by the CD56^bright^ and CD56^dim^ NK cell subsets and glucose and lipid parameters related to cardiometabolic disease. Interestingly, no significant associations were found between proteins on NK cells in blood, SAT, or VAT and HOMA-IR, as a measure of systemic insulin resistance (**Figure 3A**). However, we did observe several, mostly negative, statistically significant associations between glucose parameters and protein expression in blood, such as between the long-term glucose indicator HbA1c and CD38 on both NK cell subsets, and between glucose and NKG2D on the CD56^bright^ NK cells (**Figure 3A**). In adipose tissue, the few identified correlations with nominal significance were found with lipid parameters, such as positive correlations between both LDL and total cholesterol and CCR5 expression on the CD56^bright^ NK cells in SAT (**Figure 3A**). Given the observed associations of HbA1c and fasting glucose with NK cell proteins, we further investigated potential differences in NK cells in patients with and without T2D. However, in our material we did not find significant differences between the two groups, neither the NK cell abundance nor NK cell protein expression, which may possibly be due to the relatively low number of people with T2D in our cohort (n=9) (**Figure 3B**, **Supplementary Figure 3**). In summary, we observed some significant associations between NK cell proteins and circulating glucose- and lipid parameters, however, the weak associations with systemic insulin resistance and T2D in our cohort suggest that expression of individual proteins may not provide sufficient power to identify such link.

### High-dimensional analysis identifies subsets of adipose tissue-resident CD56^bright^ NK cells

**Figure 4:**
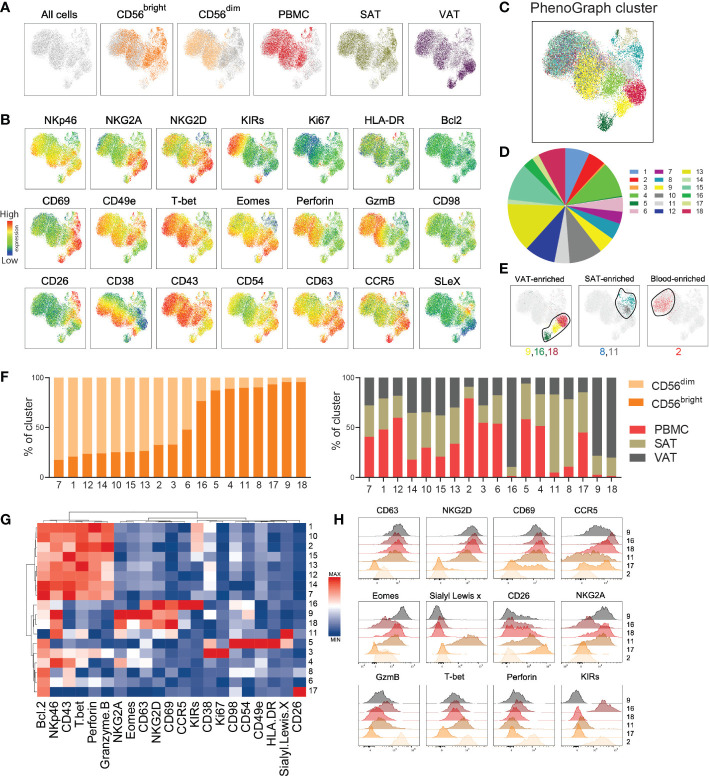
High-dimensional analysis reveals distinct NK cell subclusters. **(A)** UMAP plot showing the distribution of CD56^bright^ and CD56^dim^ NK cells from peripheral blood mononuclear cells (PBMC), subcutaneous adipose tissue (SAT) and visceral adipose tissue (VAT) of 33 individuals with obesity. The NK cell subsets from each sample were barcoded, down sampled to 5000 cells per sample and concatenated. UMAP projection colored according to the cell type and tissue of origin. **(B)** Expression intensities of 21 proteins within the UMAP plot. **(C)** UMAP plot overlaid with 18 identified PhenoGraph clusters. **(D)** Donut plot showing proportions of each cluster out of the total concatenated NK cell population. **(E)** Selected PhenoGraph clusters displayed over UMAP embeddings showing clusters enriched in VAT, SAT, and blood. **(F)** Stacked bars showing relative abundance of CD56^dim^ and CD56^bright^ NK cells (left) and tissue of origin (right) within each cluster. **(G)** Heatmap displaying Z-score transformed median fluorescence intensity (MFI) expression values for each of the proteins across the 18 clusters. Color scale is determined for each column separately, based on the lowest and highest Z-score value for that protein. **(H)** Flow histograms showing expression of proteins within the indicated clusters.

For a higher resolution depiction of NK cell diversity, we employed the high dimensional reduction technique, Uniform Manifold Approximation and Projection (UMAP), on CD56^bright^ and CD56^dim^ NK cells from blood, SAT, and VAT to identify protein combinations on a single cell level. UMAP identified multiple clusters of CD56^dim^ and CD56^bright^ NK cells deriving from the different tissue compartments (**Figure 4A**). The CD56^dim^ NK cells from both blood, SAT, and VAT primarily co-localized in two clusters, whereas the CD56^bright^ NK cells widely dispersed into multiple SAT and VAT-derived clusters, suggesting higher degree of phenotypic diversity among this subset (**Figure 4A**). The UMAP plots display variation in expression levels of each of the proteins in the flow cytometry panel across the different clusters within both the CD56^dim^ and CD56^bright^ NK cell populations (**Figure 4B**). CD38 and Sialyl Lewis^X^ in CD56^bright^ cells from VAT and SAT, respectively, and variation of KIR expression in CD56^dim^ cells from all compartments were examples of surface proteins showing high variation (**Figure 4B**). Thus, the analysis identified multiple clusters of CD56^bright^ and CD56^dim^ NK cells with different levels of protein expression, pointing to the existence of several subpopulations of NK cells.

Indeed, PhenoGraph clustering analysis identified 18 defined clusters of NK cells within the UMAP plot with various size proportions (**Figures 4C-E**, **Supplementary Figure 4**). Most clusters were enriched with either CD56^dim^ or CD56^bright^ NK cells, with the exception of cluster 6, containing equal proportion of both subsets (**Figure 4F**, left). The majority of clusters contained cells deriving from all tissue compartments (**Figure 4F**, right), but as expected, the VAT-enriched (#9, #16, #18) and the SAT-enriched (#8, #11) clusters were dominated by CD56^bright^ NK cells, and the blood-enriched cluster (#2) were dominated by CD56^dim^ NK cells (**Figure 4E, F**). A hierarchical clustering, summarizing protein expression patterns across the 18 clusters, confirmed the similarities between the VAT-enriched CD56^bright^ NK cell clusters (#9, #16, #18), all displaying considerably higher levels of CD63, CD69, and NKG2D compared with the other clusters (**Figures 4G, H**). However, differences were also observed across these clusters, such as higher levels of Eomes, NKG2A, and CD38 in #9 and CCR5 and KIRs in #16, revealing proteins that may define the different subclusters of CD56^bright^ NK cells in VAT. As expected, the clusters enriched with CD56^dim^ NK cells (e.g., #1, #2, #10) shared phenotypic signatures, such as higher levels of perforin, granzyme B and T-bet (**Figure 4G**).

Given the few associations observed between the single proteins and metabolic parameters (**Figure 3A**), we next investigated the relationship between the NK cell clusters and clinical traits. To this end, the patients were stratified into “low” and “high” based on the median value of the clinical parameters from all patients (**Table 1**). Overall, the clusters shared relatively similar distribution of NK cells deriving from “low” and “high” patients for most of the parameters, with exception of cluster #14 that contained higher proportions of cells from patients with “high” BMI and HOMA-IR (77% and 78%, respectively) and cluster #16 that contained higher proportions of cells from patients with “low” BMI, HOMA-IR, and LDL (74%, 80%, 79%, respectively) (**Supplementary Figure 5**).

Taken together, by employing high dimensional clustering analysis, we identified protein signatures defining distinct subsets of CD56^bright^ and CD56^dim^ NK cells in blood, SAT, and VAT from people with obesity. Importantly, we discovered potential subpopulations of tissue-resident CD56^bright^ NK cells, mainly residing in VAT, defined by the co-expression of CD63, CD69, and NKG2D.

## Discussion

The main function of immune cells is to combat pathogens and infections. However, it has become increasingly clear that immune cells are also involved in maintaining tissue homeostasis, mainly exerted by local immune cell populations that reside in the tissues and do not enter the circulation (14). The concept of tissue-residency has rapidly evolved over the last years, and it is now evident that also NK cells constitute a widely heterogeneous population of resident cells across various tissues (8, 15). Since the proteins that define specific NK cell populations are dependent on the tissue type, the discovery of tissue-specific markers has been important to study the human NK cell compartment during homeostatic and pathological conditions.

In adipose tissue, resident NK cells have recently gained increased interest as mediators of pro-inflammatory signaling that links obesity to insulin resistance (4, 5). Thus, targeting specific adipose tissue NK cell population to reduce inflammatory signaling may represent a future pharmacological strategy to treat obesity-related co-morbidities. However, one major shortcoming to achieve this goal is the gap in knowledge of the phenotypic markers that define NK cell subsets residing in human adipose tissue in the setting of obesity. In this study, we analyzed CD56^bright^ and CD56^dim^ NK cells in blood and two adipose tissue depots from a cohort of individuals with obesity, using a high-dimensional flow cytometry panel that combined established NK cell tissue residency-, maturation-, and effector proteins with proteins from a broad surface receptor screening. We identified NK cell subsets with unique protein signatures and distribution patterns between blood and between adipose tissue depots, thus providing novel insight of NK cell heterogeneity within the adipose tissue.

As expected, our flow cytometry data revealed that the proportion of CD56^bright^ NK cells in blood was low. Interestingly, this was also true for SAT, suggesting a low frequency of tissue-resident NK cells in this adipose depot. By contrast, the proportion of CD56^bright^ NK cells was higher in VAT, which is in accordance with previous reports and supports that obesity promotes proliferation of tissue-resident NK cells in VAT (13, 16). Moreover, since CD56^bright^ NK cells are the main cytokine-producing subset, the CD56^bright^ enrichments in VAT possibly reflects more pro-inflammatory signaling in this depot.

Phenotypic differences between the CD56^bright^ and CD56^dim^ NK cells in adipose tissue resembled the phenotypes described in other tissues. Notably, the increased levels of granzyme B and perforin on the CD56^dim^ NK cells manifested their cytotoxic potential and the elevated KIRs expression on the CD56^dim^ cells and NKG2A expression on the CD56^bright^ NK cells confirmed their distinct maturation profiles (17, 18). Furthermore, the inverse expression patterns of CD69 and CD49e on the CD56^bright^ cells supported the tissue residency nature of this subset (19). Tissue-resident CD56^bright^ NK cells may also be shaped by the tissue microenvironment and can be characterized by the expression of distinct tissue-specific proteins (8, 14). In the liver, such adaptations have been reflected by the identification of CXCR6, CCR5 and Eomes on resident CD56^bright^ NK cells (20, 21), whereas NK cells residing in the uterus express CD9 (22). In our study, we identified increased levels CD26, CD54, CD63, CCR5, and Sialyl Lewis^x^ on the resident CD56^bright^ NK cells in adipose tissue, with most proteins found enriched in VAT compared with SAT. Several of these proteins are linked to activation and have previously been shown to be expressed by tissue-resident NK cells in other organs, including the chemokine receptor CCR5 in the liver (20) and the adhesion molecule CD54 in lymphoid tissues (23). Also enriched on adipose CD56^bright^ NK cells in the adipose tissue was the metabolic protein CD26, previously shown upregulated on NK cells upon activation to facilitate cytokine production (24), and the exosome marker CD63, which has been related to NK cell effector functions (25). To what extent these proteins play an active role in tissue retention or execute other relevant functions on the tissue-resident CD56^bright^ NK cells remains to be established. It should be noted that the lack of CD127 in our gating strategy prevents us from formally excluding ILCs from the CD56^bright^ NK cell population. However, since the majority of CD56^bright^ cells were positive for Eomes, a protein not expressed by ILCs, we can assume that the CD56^bright^ NK cell population is primarily made up of bona fide NK cells. Our data suggests that CD56^bright^ NK cells share similar phenotypic features across different tissues, although the relative expression of the different surface proteins may vary, possibly depending on differences in the microenvironment (7). The differences in protein expression that we observed between SAT and VAT may reflect adaptations by such microenvironmental signals, including differences in levels of hormones, fatty acids, and adipokines, that are known to fluctuate in the adipose tissue to meet increased energy requirements in obesity (26). Structural differences between SAT and VAT, such as the extracellular matrix (ECM) composition, may potentially also influence the immune cell phenotypes within the distinct depots (27). The UMAP and PhenoGraph analyses indicated the existence of multiple adipose tissue NK cell clusters, underscoring recent evidence of NK cell heterogeneity in adipose tissue (28). In our analysis, VAT was enriched with clusters of resident CD56^bright^ NK cells defined by the co-expression patterns of CD69, CD63, and NKG2D, with CCR5 and Eomes defining specific subclusters. Although their functional relevance requires further evaluation, the observed co-expression of these proteins might define specific NK cell subsets relevant for inflammation in VAT and potentially for the development of insulin resistance.

A relationship between NK cells and insulin resistance have been reported by several (11, 29). However, such associations could not be found in our cohort when examining the individual proteins and by using HOMA-IR as a measure of insulin resistance. There may be several explanations for this lack of association. Firstly, it should be noted that our analyses included biological material from patients with class III obesity only, and inclusion of lean controls may have given a different result. Also, HOMA-IR does not provide the same accuracy as an hyperinsulinemic-euglycemic clamp measurements, considered the gold standard of insulin resistance measurement (30). Furthermore, HOMA-IR measures systemic insulin resistance and does not reflect insulin resistance in adipose tissue, which ideally requires measurements of adipose tissue lipolysis (31). In addition, it is important to acknowledge the fact that pro-inflammatory immune cells should not merely be considered pathological, since they also have protective effects in the obese adipose tissue. This was recently demonstrated for a subset of crown-like structure, pro-inflammatory macrophages that were in fact essential for adipose tissue homeostatic functions during nutrient excess (32). Thus, the co-existence of pro-inflammatory adipose tissue immune cell subtypes with potentially both beneficial and dysregulatory functions may, at least partly, disrupt potential correlations with the metabolic parameters.

Taken together, we have performed a high dimensional profiling of NK cells in blood and adipose tissue in human obesity and identified depot-specific proteins defining adipose tissue-resident NK cell subsets. The observed heterogeneity of NK cells residing in adipose tissue underlines the importance of identifying such proteins to distinguish between subsets contributing to metabolic homeostasis and subsets mediating the pathological signaling related to metabolic dysfunction.

## Material and methods

### Clinical cohorts

Several clinical cohorts were included in the current study. This was approved by the Regional Committees for Medical and Health Research Ethics in Bergen (REK: 2010/502 and 2015/2343) and written consent was obtained from all participants. First, subcutaneous adipose tissue from liposuction aspirates of individuals undergoing plastic surgery was used for proteomic screening. Buffy coats from anonymous blood donors were used as blood samples and internal controls for the cell surface screening. For the 27-color flow cytometry analysis, matched fasting blood samples and subcutaneous- and visceral adipose tissue biopsies were obtained from 43 patients with obesity undergoing bariatric surgery at Voss Hospital. The clinical characteristics and biochemical measurements of this cohort are presented in **Table 1**.

### Isolation of stromal vascular fraction and peripheral blood mononuclear cells

Subcutaneous adipose tissue and visceral adipose tissue biopsies obtained from bariatric surgery were immediately stored in Krebs-Ringer Phosphate (KRP) buffer until processing. The biopsies were cut into pieces and enzymatically digested with collagenase Type I (Life Technologies) for 1 hour at 37°C with constant shaking. The subcutaneous adipose tissue from liposuction was washed in 0.9% NaCl, diluted in KRP buffer and digested with Liberase (Roche). The dissolved tissues were filtered, and the stromal vascular cells (SVC) were isolated from the mature adipocytes and washed with Phosphate Buffered Saline (PBS). The liposuction SVC was freshly stained, whereas the SVC from biopsies were preserved in freezing media containing FBS and 10% DMSO and stored in liquid nitrogen until further flow cytometry experiments. Peripheral blood mononuclear cells (PBMC) were isolated from blood of bariatric surgery patients and healthy donors using density gradient centrifugation, as previously described (33).

### Surface proteomic screening

LEGENDScreen™ Human PE kit (Biolegend, Cat#700007) was used to screen the cell surface proteome. Fresh SVC from subcutaneous liposuction aspirates and PBMC from buffy coat blood were used in the screening as previously described (33). In brief, the tissue-derived and blood-derived cells were CD45 barcoded and stained with a backbone antibody panel allowing identification of the cells of interest. The cells were added to the plates provided in the LEGENDScreen™ kit and the staining was performed according to the manufacture protocol. Plates were run on an 18-color flow cytometer (LSR Fortessa, BD Biosciences) with 407, 488, 561 and 640 lasers using the BD FACSDiva™ Software (BD Biosciences). Flow cytometry data was analyzed using FlowJo v10 (Treestar, USA). Two manual gating approaches were performed to identify the NK cell subsets, a “broad” gating followed by a “fine” gating. Several adipose-enriched proteins identified from both gating approaches were validated for expression in additional adipose tissue samples, and a selection of these proteins was included in the 27-parameter flow cytometry experiment.

### 27-parameters flow cytometry staining

Frozen PBMC and SVC were thawed and stained with an extracellular primary antibody panel containing the following antibodies from BD Biosciences: BUV496 CD16 (Cat# 612944), BUV563 CD56 (Cat# 565704), BUV661 CD38 (Cat# 612969), BUV737 CD69 (Cat# 612817), BUV805 CD45 (Cat# 612891), BB700 NKG2A (Cat# 747926), APC Vio770 CCR5 (Cat# 557755), V500 CD14 (Cat# 561391), BV510 CD19 (Cat# 562947), BV650 CD98 (Cat# 744505), BV711 Sialyl Lewis x (Cat# 563910), PE-Cy5 CD54 (Cat# 555512). From Biolegend: Biotin NKp46 (Cat# 331906), A700 CD63 (Cat# 353024), BV421 Bcl-2 (Cat# 658709), BV510 CD123 (Cat# 306022), BV750 CD3 (Cat# 344845), BV785 HLA-DR (Cat# 307642), PE CD26 (Cat# 302706), PE-Cy7 NKG2D (Cat# 320812), PE-Cy7 CD162 (Cat# 328816), BB515 CD49e (Cat# 130-110-534, Miltenyi), PE-Cy5.5 KIR2DL1/S1 (Cat# A66898, Beckman Coulter), PE-Cy5.5 KIR2DL2/L3/S2 (Cat# A66900, Beckman Coulter). Staining was performed in FACS buffer (PBS with 2mM EDTA (Cat# AM9260G, Ambion), 2% FCS (Cat# F7524, Sigma). After 20 min incubation at room temperature (RT) and in the dark, the cells were washed twice with FACS buffer and further stained with a secondary antibody panel containing streptavidin (Cat# 624294, BD Biosciences) for 15 min at RT in dark. Cells were washed twice with FACS buffer before adding Fix/perm Buffer (diluted ¼ with eBioscience reagents: Fix/perm diluent (Cat#00.5223.56) and Fix/perm concentrate (Cat#00.5123.43)) and incubated for 45 min RT in dark. Cells were then washed twice with perm/MQ buffer (Perm Buffer 10X (Cat#00.8333.56) diluted 1/10 with MQ water). Further, the cells were stained with an intracellular antibody panel containing the following antibodies from BD Biosciences: BUV395 Ki67 (Cat# 564071), BB755-P Perforin (Cat# 624391, BD Horizon custom reagents), BB790-P Granzyme B (Cat# 624296, BD Horizon custom reagents), and eF660 Eomes (Cat# 50-4877-41, eBioscience), PE-Dazzle594 T-bet (Cat# 644828, Biolegend). Live/dead Fixable Aqua Dead Cell stain kit (Invitrogen, 1:100 dilution) was used to distinguish between dead and live cells. After 30 min incubation, the cells were washed twice with perm/MQ buffer and kept in FACS buffer for immediate flow cytometry analysis. Samples were run on a 29-color Symphony (BD Biosciences) with 405, 488, 561 and 639 lasers using the BD FACSDiva™ software (BD Biosciences). Flow cytometry data generated was analyzed using FlowJo V10 (Treestar, USA).

### Dimension reduction analysis

The FlowJo plugins Uniform Manifold Approximation and Projection (UMAP) and PhenoGraph were performed to visualize the single cell flow cytometry data in a high dimensional structure. To this end, CD56^bright^ and CD56^dim^ NK cells from PBMC, SAT, and VAT for each patient (n=33) were electronically barcoded for cell subset, tissue of origin and several biochemical parameters (either “low” or “high” based on median values of each parameter). The patient samples containing the same cell subset and tissue type were concatenated (6 samples in total), which was downsampled to 5118 events/file for an equal number of cell input. The samples were concatenated to a final file that was analyzed for UMAP and Phenograph. UMAP was run using the default settings (Euclidean distance function, nearest neighbors: 15 and minimum distance: 0.5). PhenoGraph was run using the default number of nearest neighbors (K = 30). The parameters included in both analyses were the phenotypic markers of interest (n=21) and excluded the proteins used to gate on the NK cell subsets.

### Statistical analysis

Flow cytometry data was analyzed using Prism version 9.2.0 (GraphPad). D’Agostino & Pearson omnibus normality test was used to determine normality of the data. For normally distributed data, one-way ANOVA with Holm-Šídák’s multiple comparisons test was used. When data was not normally distributed, Friedman test with Dunn’s multiple comparisons test was used. A p-value of < 0.05 was considered statistically significant. Correlation analysis was performed in R using the Pearson correlation coefficient and considered significant at adjusted FDR <0.1.

## Data availability statement

The original contributions presented in the study are included in the article/**Supplementary Materials**. Further inquiries can be directed to the corresponding author.

## Ethics statement

The studies involving human participants were reviewed and approved by Regional Committees for Medical and Health Research Ethics in Bergen (REK: 2010/502 and 2015/2343). The patients/participants provided their written informed consent to participate in this study.

## Author contributions

MEH designed panel, planned and performed experiments, acquired and analyzed data and wrote the manuscript. MC designed panel, planned experiments, analyzed data and reviewed/edited the manuscript. KS planned and performed experiments and analyzed data. NS planned experiments. DS and LL-A analyzed data. IDH acquired data. CB sampled adipose tissue. GM contributed to the discussion and reviewed/edited the manuscript. NB and JF designed the study, oversaw its conduction, provided funding, contributed to the discussion and reviewed/edited the manuscript. All authors contributed to the article and approved the submitted version.
