# Supplementary material for: Phenotypic diversity of human adipose tissue-resident NK cells in obesity
Source: Front Immunol. 2023 Feb 22;14:1130370. doi: 10.3389/fimmu.2023.1130370 (PMC9996326; doi:10.3389/fimmu.2023.1130370)
Supplement: Supplementary file 1 [file DataSheet_1.docx]

**Supplementary figures**

**Phenotypic diversity of human adipose tissue-resident NK cells in obesity**

Martha E. Haugstøyl, Martin Cornillet, Kristina Strand, Natalie Stiglund, Dan Sun, Laurence Lawrence-Archer, Iren D. Hjellestad, Christian Busch, Gunnar Mellgren, Niklas K. Björkström, Johan Fernø

**Supplementary Figure 1: Protein expression levels on NK cell subsets.** Box-and-whisker plots depicting the mean fluorescence intensity (MFI) of proteins on CD56^bright^ and CD56^dim^ NK cells in PBMC, SAT and VAT of individuals with obesity (n=43). Line represents median, and the whiskers represent the minimum and maximum value.


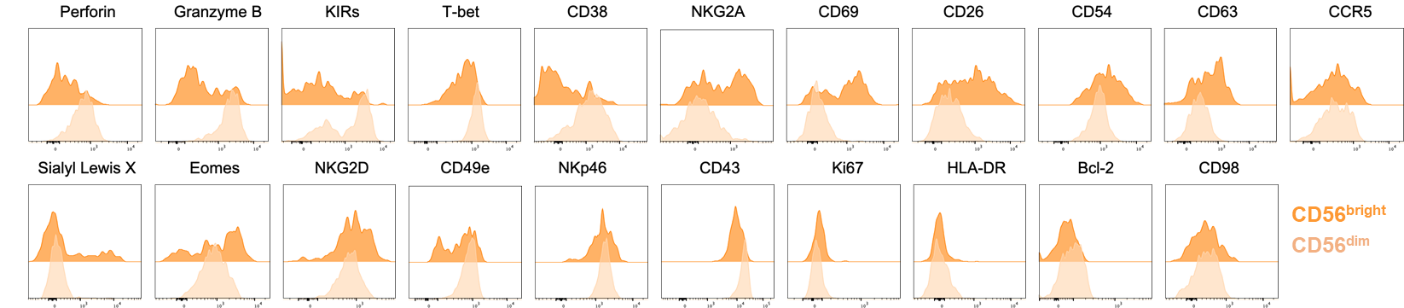


**
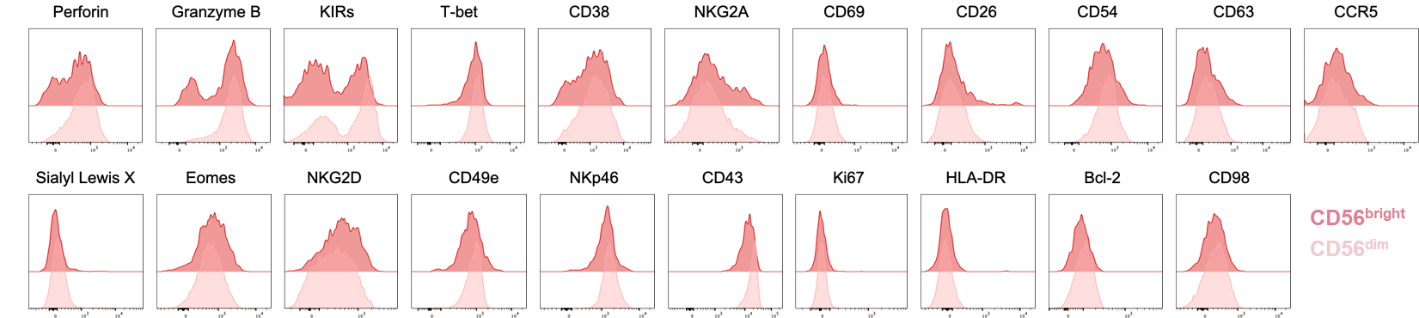
**

**Supplementary Figure 2: Representative stainings of NK cell proteins.** Histograms showing modal marker expression intensity on CD56^bright^ and CD56^dim^ NK cells in subcutaneous adipose tissue (orange) and peripheral blood (red) from one patient.

**Supplementary Figure 3: Protein expression on NK cell subsets in T2D.** Box-and-whisker plots depicting the percentage of CD56^bright^ and CD56^dim^ NK cells expressing the indicated proteins in PBMC, SAT and VAT of individuals with obesity with T2D (n=34) and without T2D (n=9). Line represents median, and the whiskers represent the minimum and maximum value.

**
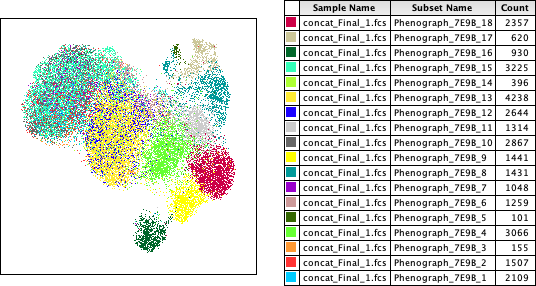
**

**Supplementary Figure 4: Phenograph NK cell clusters.** Overview of 18 Phenograph clusters from concatenated CD56^brigth^ and CD56^dim^ NK cells from PBMC, SAT and VAT (n=33).

**
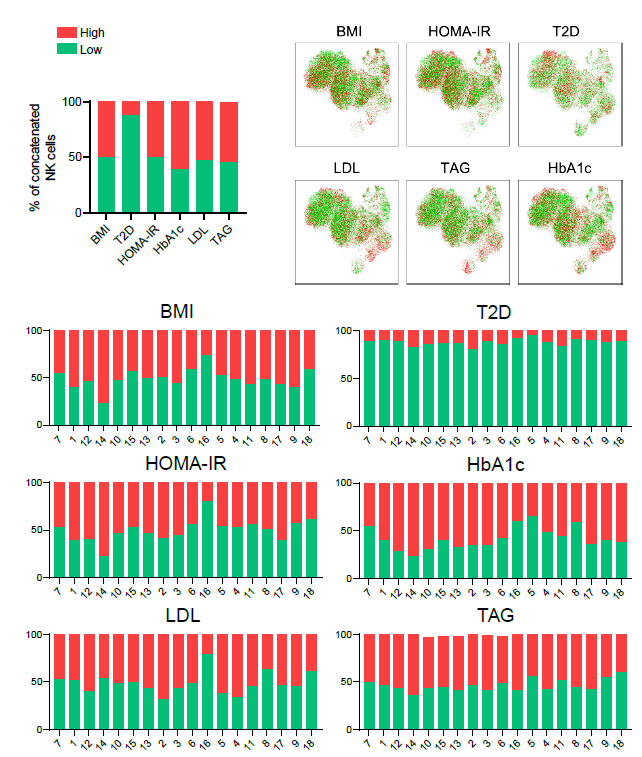
**

C

B

A

**Supplementary Figure 5: Clinical traits of Phenograph clusters. (A)** Proportions of concatenated NK cells from individuals with obesity (n=33) labeled as either “low” or “high” of various clinical parameters based on the median values of all patients. T2D parameter is classified as “no” (green) or “yes” (red). **(B)** UMAP plot showing the distribution of NK cells labelled as either “low” or “high” for each parameter. **(C)** The distribution of NK cells labelled as either “low or high for each parameter within each Phenograph cluster.
